# Evaluating the Healthy Futures Nearby Program: Protocol for Unraveling Mechanisms in Health-Related Behavior Change and Improving Perceived Health Among Socially Vulnerable Families in the Netherlands

**DOI:** 10.2196/11305

**Published:** 2019-04-02

**Authors:** Lette Hogeling, Lenneke Vaandrager, Maria Koelen

**Affiliations:** 1 Health and Society Department of Social Sciences Wageningen University and Research Wageningen Netherlands

**Keywords:** health behavior, socioeconomic status, health promotion, healthy lifestyle, Netherlands, inequalities

## Abstract

**Background:**

The persistence of health inequalities within high-income societies such as the Netherlands indicates the importance of researching effective ways to reduce those inequalities. Multiple strategies for reducing health inequalities have been identified. Specifically targeting health-related behaviors among lower socioeconomic status groups is one of those strategies. All in all, it seems relatively clear what types of approaches in general lead to health-related behavior change. However, it is still unclear *how* these approaches, in interaction with context, trigger a specific desired change. In the Netherlands, the private funding organization, Fonds NutsOhra, funded 46 small-scale projects under the umbrella of the Healthy Futures Nearby program. The projects aim to reduce vulnerable families’ health deprivation by triggering lifestyle changes.

**Objective:**

This study aimed to outline and justify the protocol for the overall evaluation of the program. The evaluation aimed to find out *to what extent* and *how* the small-scale projects and approaches within the program affect (or not) health-related behaviors and improve perceived health.

**Methods:**

The approach to the overall evaluation of the 46 projects builds on a combination of 3 frequently used evaluation models; it is theory-based, realist informed, and uses a mixed methodology design. Methods include analysis of quantitative project data, document analysis, focus groups, and interviews. A study design has been drawn up that values and uses the multifaceted development of the projects and the influence this might have on implementation and project outcomes. Also, it respects the complex nature of the projects and is suited to studying health promotion mechanisms in depth. Finally, it optimizes the usage of all—quantitative and qualitative—project evaluation data available.

**Results:**

This study protocol included the design of at least 4 different studies. The results will hence provide information on (1) building and defining theories of change in health promotion practice, (2) mechanisms at work in promotion of healthy behavior among vulnerable families, (3) what works and what does not in professionals’ practices in health promotion among those vulnerable groups, and (4) what works and what does not in health promotion projects with a participatory approach. In addition, data will be collected on the overall effectiveness of the 46 initiatives. Data collection started in 2016. Data analysis is currently underway, and the first results are expected to be submitted for publication in 2019.

**Conclusions:**

This overall evaluation provides a unique opportunity. The diversity of projects allows for a study protocol that answers in greater depth questions of *how* specific health promotion approaches work while also elucidating their effectiveness in a more traditional way. Using a theory-based complexity-sensitive approach that is mainly realist informed, this study also provides an opportunity to see whether combining assumptions from different evaluation perspectives yields relevant information.

**International Registered Report Identifier (IRRID):**

DERR1-10.2196/11305

## Introduction

### Background

In the Netherlands, life expectancy has increased over the past decades. The National Institute for Public Health and the Environment also reports an increase in *healthy* life expectancy [1], meaning that people are not only living longer but also living longer *healthier* lives. However, inequalities in health between and within countries—including high-income countries such as the Netherlands—remain substantial [2,3]. Health inequalities are an issue of fairness and social justice [4,5]. People who are vulnerable to health deprivation may not reach their full potential as individuals and as a group in society. The issue is even more pressing as health inequalities appear to be reproduced from one generation to the next [6,7]. The persistence of health inequalities within societies indicates the importance of research on what the World Health Organization (WHO) has called the *social determinants* of health [8] and on policies that aim to reduce inequalities. There have been many studies on the causes of health inequalities, both within and outside the Netherlands [2,9-12].

Besides looking at what causes health inequalities, scholars, policymakers, and practitioners have dedicated themselves to finding ways to reduce those inequalities. In the Ottawa Charter, WHO stated [13], and more recently the International Union for Health Promotion and Education declared in its Curitiba statement [14], that addressing the social, environmental, and economic determinants of health is crucial for reducing health inequalities, in addition to recognizing the importance of personal skills and capabilities [15,16]. Furthermore, WHO has stated the importance of involving a range of stakeholders, including citizens, in health promotion initiatives. Multiple strategies for reducing health inequalities have been identified [5,17-19], of which specifically targeting health-related behavior among lower socioeconomic status groups is one. In the Netherlands, Beenackers et al [20] conducted a review on effective interventions for behavioral change leading toward the reduction of health inequalities, focusing on smoking, alcohol consumption, overweight, and perceived health. Overall, they concluded that approaches could be more effective in changing behavior if they are targeted specifically at the needs of those vulnerable to deprivation, if they use existing structures and the expertise of local health professionals, and if they are designed in an integrated way; this means including various perspectives and involving different sectors and stakeholders. Others have written about the effectiveness of a community-based approach in reducing health inequalities. However, for each of these measures, substantial uncertainties remain around successful implementation [21,22]. Contextual factors appear to have a major influence on whether specific approaches, or elements of approaches, work or not. Community-based approaches work in some cases but have proved much less effective in others [21,23]. Successful collaboration with local experts may be largely dependent on whether such a network actually exists, whether professionals are open or willing to collaborate, whether previous local projects have been successful and thus what the initial starting position is, and so on. All in all, even though it may be relatively clear what types of approaches in general lead to better health, it is still unclear how certain approaches, or elements of approaches, in interaction with context trigger specific outcomes.

More traditional approaches to health promotion evaluation focus predominantly on researching evidence for specific interventions by measuring (quantitatively) the effectiveness of predefined outcomes. However, evidence on the effectiveness of interventions does not provide a sufficient or workable base for future work in health promotion. As argued, varying and dynamic contexts combined with participatory approaches require in-depth study of mechanisms rather than of specific interventions. What mechanisms underlie successes in the promotion of healthy behaviors or the discouragement of health risks? What contexts enable or hinder such processes? To answer such questions, more in-depth studies and data are needed to enable researchers to look at different social and physical settings and mechanisms at play within those contexts. These studies should be designed to grasp the full interactions, relations, and influences of and between contextual factors, interventions, mechanisms, and outcomes. This paper outlines the protocol for an evaluation study particularly aimed at unraveling these mechanisms. We have made an effort to create an overall evaluation plan that does justice to the dynamics and complexity of local, community health promotion projects and results in relevant information on what works in (the process of) promoting a healthy lifestyle. The novelty of our design lies, among other things, in our flexible approach to evaluation with regard to the initiatives’ plans and dynamics, creativity in collecting and combining diverse data, and the focus on *what* works instead of *which project* works.

### Research Questions

A program (Textbox 1) funding 46 small-scale health promotion projects within the Netherlands [24] presented the opportunity to study what happens in different settings and contexts while also looking in depth at processes at play. These diverse, merely local initiatives have all designed their own intervention and evaluation. Information from these initiatives and evaluations is available for the overall evaluation. The combination of diverse small-scale projects offers both a very broad and an in-depth source of information on the workings of health promotion through lifestyle changes in specific contexts. This provides a unique opportunity to study mechanisms for changing socially vulnerable families’ health-related behaviors. The overall evaluation aims to find answers to 2 main questions:

1. To what extent do (shared) approaches within small-scale projects affect health-related behaviors and improve perceived health (impact)?
2. How do the approaches within the program affect (or not) health-related behaviors and improve perceived health (mechanisms)?

### Opportunities

There are 4 ways in which the program and projects (Textbox 1) offer a unique opportunity to study health promotion mechanisms: (1) the evaluation data from the 46 small-scale projects potentially enable the study of the projects’ *effectiveness* in changing health-related behavior and improving perceived health; (2) the projects offer a diverse and in-depth source of information on how particular approaches, in different contexts, may lead to specific outcomes and thus provide a basis for *unraveling mechanisms* of health promotion; (3) partial homogeneity in approaches and desired outcomes provides the opportunity to *compare* the effectiveness and mechanisms of similar approaches in varying contexts; and (4) the timing of the overall evaluation, parallel to the implementation of the projects, allows for a strong focus during the evaluation on *learning while doing* for all stakeholders involved. These 4 opportunities, elaborated below, set the framework for the overall evaluation.

**The Healthy Futures Nearby program and projects.**

In the Netherlands, the private funding organization, Fonds NutsOhra, funded 46 small-scale projects under the umbrella of the Healthy Futures Nearby (HFN) program [24] and issued an overall evaluation of the program to learn about participation, effectiveness, and sustainability. The projects all aim to reduce health inequalities through lifestyle changes in vulnerable families.

The HFN program aims to “improve the health behaviors of vulnerable families with a low socioeconomic background to reduce health inequalities.” Vulnerable families are defined as households in which at least 1 adult and 1 child live together, who experience multiple problems with finances, education, work, or well-being and who suffer health deprivation by smoking, heavy consumption of alcohol, or unhealthy weight combined with a lower perceived health.

- Projects have been awarded funding for the years 2016 to 2019 (34 projects) or 2017 to 2019 (12 projects).
- Projects use either a neighborhood-oriented (similar to community development) participatory approach or work from an intersectoral approach (similar to a systems perspective, including different stakeholders and levels) to reduce inequalities by promoting healthy lifestyles. These 2 approaches can be understood as the program’s *theory of change*.
- All 46 small-scale projects focus directly or indirectly on reducing alcohol use, promoting smoking cessation, promoting a healthy weight and improving perceived health. To reach their goals, the projects develop and implement a range of strategies and activities. For instance, some employ a participatory, dynamic neighborhood-oriented approach, whereas others focus on improving social infrastructures for families facing multiple problems.

**Figure 1:**
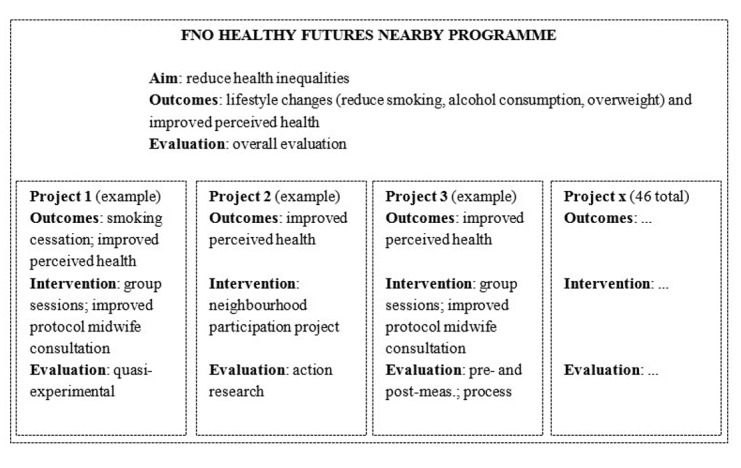
Structure of the Fonds NutsOhra (FNO) Healthy Futures Nearby program.

All projects conduct their own project evaluations, which almost always include pre- and postmeasurement of project-specific outcome measures (behaviors and perceived health) among vulnerable families (Figure 1).

First, the information made available by the projects is potentially of high enough quality to examine to some extent the effectiveness of their activities and approaches. Such information on effectiveness could provide relevant information at the higher program or policy level [25]. Projects conduct their own evaluations, consisting of at least a baseline and post activities measurement of relevant project-specific outcomes at the participant (family) level. However, project-specific research designs vary greatly and not all projects use a (quasi) experimental setup, meaning that for instance control groups are not included in most of the project evaluations. Considering these limitations, by examining effectiveness, we aimed to study the extent to which the projects have changed health-related behaviors and led to an improved perceived health among socially vulnerable families participating in the projects. An initial exploration of the combined project baseline data will help determine which methodological approach is best suited to combine and analyze the set of information on health-related behavior.

A second opportunity lies in the in-depth information contained in the 46 small-scale projects, which can potentially be very useful for unraveling mechanisms. Whereas effectiveness is often the central focus of evaluation, the diversity of projects under study here is suited to answering more in-depth questions on how the various health promotion approaches work (or not) in different contexts. Various projects work with similar approaches, enabling the study of these approaches in different contexts. Understanding the influence of contextual factors—social, historical, and physical—has been identified as crucial to policymakers’ and practitioners’ successful implementation of health promotion initiatives [26]. To generate success, it is essential to understand under what circumstances specific interventions may work or not. Evaluation should aim to generate knowledge on these context-mechanism interactions instead of focusing solely on the experimental effectiveness of interventions. Context should therefore play a major role in learning through evaluation. Also, the main challenge in learning from (community) health promotion projects is to study and define *how* and *why* communities may benefit [21]. This has often been addressed as opening the black box: it is known whether a specific project works or not, but possibilities for transferability are limited because it is not known *why* and *how* the project or approach works or does not work in relation to a specific context [27]. The 46 projects can provide that in-depth information. Looking at mechanisms and including contextual influences does, however, have implications for the evaluation design. More traditional, experimental evaluation designs do not suffice. Although the interaction between approach and context is considered an important factor in health promotion projects, designs such as randomized controlled trails deliberately exclude such contextual influences to keep causal relations *clean*. Also, these designs leave little room for variations in approaches, dynamics, and changes during implementation and valuable unexpected effects and serendipity—all very relevant in the complex reality addressed in health promotion interventions. Kok et al [22] provided 6 reasons why a more traditional, reductionist approach is not well-suited to health promotion evaluation: lack of clarity about what the approach precisely is; lack of clarity about what is expected of local contexts for effectiveness; the very diverse and open settings for health promotion; diversity in organizations and underdeveloped organizational systems; the impossibility of realizing similar configurations in different locations; and the difficulty in determining whether a project or approach works in practice as intended.

Third, the information from the projects is sufficiently diverse and substantial to look at different approaches to changing health-related behavior: promoting healthy behavior or discouraging risky behavior. Groups of projects work toward a similar outcome in varying ways, such as promoting smoking cessation (outcome) by offering one-on-one counseling or organizing group sessions (approaches). Also, some projects appear to be working along the lines of similar approaches but aim at different outcomes—for instance, the development of a participatory project together with neighborhood residents to raise awareness of risky health-related behaviors or to increase active citizen participation in neighborhood activities. The existence of similar approaches and outcomes within projects allows us to additionally compare groups of projects.

A fourth opportunity resides in the fact that the development and evaluation of the 46 projects are themselves relevant processes. A range of different stakeholders have been and will be involved in development, implementation, and evaluation. Almost half of the project designs imply the dominance of community participation. Most projects have been inspired and shaped by policy, science, and practice. Also, timewise, the overall evaluation will be conducted parallel to the implementation of the projects. These 2 characteristics, participation of diverse stakeholders and timing of the evaluation, mean that the overall evaluation could be very much a *learning* opportunity for all involved.

## Methods

### Study Design

For the overall evaluation of the 46 projects, a protocol was designed that respects the criteria set by the program. It takes into account the multifaceted development of the projects and the influence this might have on implementation and project outcomes, and the complex nature of the program and projects [25]. The aim of the protocol was to enable researchers to study mechanisms of health promotion in depth. Finally, the design sought to optimize the usage of all—quantitative and qualitative—project evaluation data available.

The 4 opportunities, or program and project characteristics, mentioned in the previous paragraphs guided the study design. In addition, the program’s main principles that have guided the design of projects shaped the evaluation design: promotion of healthy lifestyles to reduce health inequalities, a participatory approach, an intersectoral design, and a community development approach. The evaluation design should fit these project design guidelines, if only to ensure that the potential of the information offered is harnessed. In addition, we believed that research in health promotion should ideally be oriented toward also improving practices in health promotion [28]. The methods selected for evaluation should furthermore be most likely to illuminate relevant issues, both success factors and barriers, within projects and programs and be sufficiently diverse to reflect the individual, social, cultural, organizational, and economic factors at play [28]. The overall evaluation of the 46 projects builds on a combination of 3 frequently used approaches to evaluation; it is theory-based, realist informed, and uses a mixed methodology design. We recognized the complex nature of the health promotion projects by combining these approaches. In the study, a theory-based perspective on evaluation provided opportunities to involve views from all relevant stakeholders, including those who offer more practical experience and knowledge (professionals) and those who offer knowledge from the lived experience (target group), as well as stakeholders who offer a more scientific, more abstract, or theoretical view (researchers). The theory-based perspective is important throughout the study. Frequent updating of the project theories will remind the researchers to maintain an open view on the dynamic nature of the projects’ settings, contexts, and activities. As the study was realist informed, the realist perspective was used to guide the in-depth search for mechanisms by means of realist case studies [29].

The overall evaluation will be conducted by a team of researchers from 3 organizations: Wageningen Economic Research, the Verwey-Jonker Institute, and Wageningen University, Chairgroup Health and Society.

**Figure 2:**
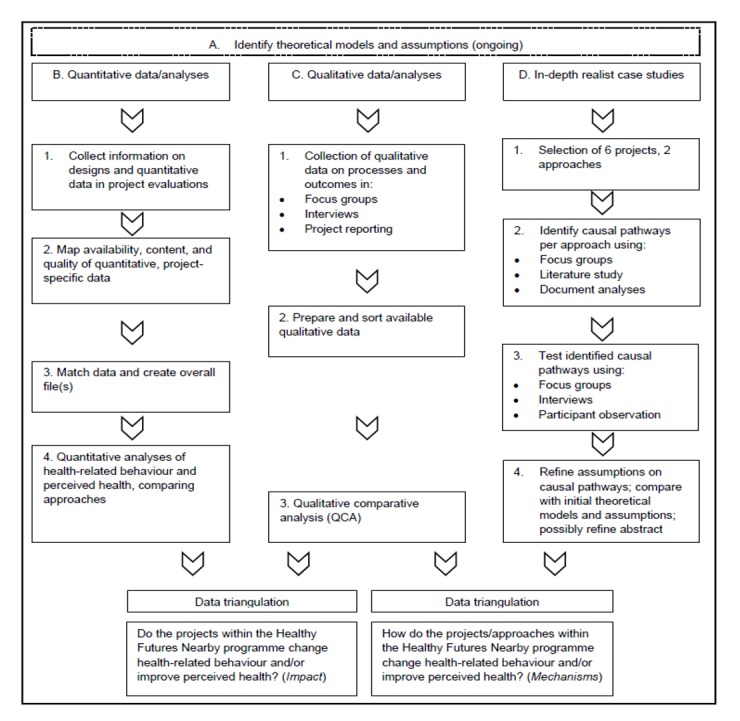
Steps in the protocol to unravel effects and mechanisms in the Healthy Futures Nearby program.

The study design encompassed 4 steps: A to D, presented in Figure 2. A is an ongoing first step to identify the theoretical assumptions about—not necessarily linear—causal pathways underlying each project. After that, step B is performed to measure effectiveness, and steps C and D are performed to study and unravel mechanisms. Each step is used to support, provide feedback into, and verify the other steps in the design. In combination (data triangulation), all steps lead to answers to both the main research questions. Each step is discussed in detail in this section.

### Step A: Identify Theoretical Models and Assumptions

The first step in the overall evaluation is to identify the (theoretical) assumptions on causal pathways for each project. Identifying and using these assumptions (theory-based evaluation) can strengthen program design and implementation and promote policy and practice learning about the effectiveness of interventions [30]. The assumptions about pathways that lead to desired outcomes in a project have been referred to in many different ways [25,31]. In this paper, we use *theoretical models and assumptions* and *presumed causal pathways* interchangeably. Following Rogers [31], both refer to “a variety of ways of developing a causal model linking program inputs and activities to a chain of intended or observed outcomes, and then using this model to guide evaluation.” The models will be identified using project proposals and, additionally, group interviews in an *Effectenarena* format [32]. This interview format is designed to facilitate participatory decision making through multistakeholder discussions. The method uses a few key concepts to streamline the group discussion: investors (stakeholders that *invest* time, money, and knowledge in the project), expected activities and conditions for those activities, and expected effects and *collectors* (those who benefit in any way). It enables the researchers as well as the project stakeholders to gain more insights into the desired outcomes, assumed causal pathways, contextual factors, and possible drivers and barriers. Focusing solely on project proposals may provide a biased result (because they are often written by project leaders). Specifically, for this study, the group interview ensures that all stakeholders involved *have their say* when it comes to assumptions relevant to the project. A group session in the *Effectenarena* format will thus be organized with each project, involving all relevant stakeholders including the vulnerable target group when possible. Two researchers will facilitate the discussions and draft a short report of the meeting, which will then be presented to the respective project leader for approval. In addition, the researcher involved will draw a visualization of the model for each project. The research team will use the sessions and reports and any other relevant documentation such as project proposals to extract for each project the underlying theoretical models and assumptions. This will be both a list of assumptions and a visual map. Given the complex, dynamic, and not necessarily linear nature of the projects, initial theoretical models and assumptions will serve as a basic set of assumptions that are open to adjustments as the projects develop. Over the course of the years, regular monitoring through interviews, group sessions, and administrative reports will build on and test these initial sets of assumptions. Insights into how HFN program principles (Textbox 1) have translated into project models and assumptions may offer valuable lessons for the implementation of future health promotion policies.

### Step B: Quantitative Data Collection and Analysis

All projects (taking place over the years 2016 to 2019) will conduct their own project-specific (primary) analysis and evaluation. Step B, in this study, includes the *overall* collection and secondary analysis of quantitative project-specific data throughout 2016 and 2019 to study program impact. Complete project-specific data and publications on desired outcomes among vulnerable families will only be available during the last stage of the overall evaluation. Also, the project-specific evaluations and the overall evaluation will be conducted in parallel. This may offer opportunities for collaboration and mutual learning, but it also requires careful planning to avoid heavy participant burden. As already mentioned, all 46 projects have their own specified evaluation design. From these project evaluations, we aim as much as possible to use the information already gathered. More specifically, our focus will be on data on perceived health and health-related behavior outcomes at the individual level of vulnerable family members. Although the projects’ evaluation designs range from randomized controlled trails to participatory action research, most conduct some form of pre- and postmeasurement of these health-related behavior outcomes. In total, 4 main activities have been distinguished in this step:

1. Collection of information on designs and quantitative data in project evaluations. Project proposals, available research proposals, and *Effectenarena* sessions will be used to collect information on the quantitative data collected in each project, by whom, for which specific target group, and using which methods and instruments. This step also includes an exploration of possibilities to combine and match data from different projects.
2. Map content and quality of quantitative project-specific data. A substantial number of project baseline measurements will take place over the first 1.5 years of the funding period. After this, we shall gather the data available from these baseline measurements. An initial exploration of the quality of the datasets will be conducted in close consultation with the (local) researchers involved in the projects.
3. Match data and create overall file(s). Statistical software R [33] will be used to match project data and thus create the overall file(s). The matching exercise will examine possible effects and compare those effects on health-related behavior and perceived health between *approaches*. Theoretically and ideally, this should lead to a number of files combining information from different projects on perceived health, weight, physical activity, smoking, and drinking behavior. Exploration of the available data as described in steps 1 and 2 will show which comparisons are possible at which levels of aggregation.
4. Quantitative analyses. Statistical software (R) will be used to compare projects and approaches relating to effects on health-related behavior and perceived health.

### Step C: Qualitative Data Collection and Analysis

A parallel step (C) in the design concerns the collection and use of qualitative data. This step entails primary data gathered by the researchers on the overall evaluation from each individual local initiative. The qualitative data will provide information to direct and support the quantitative analysis and contribute to answering *which* questions about mechanisms. In total, 3 sources of information will be included:

1. Information gathered in 46 group interviews— *Effectenarena* sessions as well as 2 *audit* sessions per project, scheduled at half term and at the end of the subsidiary period. The aim of the audits is twofold: to provide the researchers with information on outcomes and (preliminary) results, processes, and developments in the different projects, while creating space for project teams to reflect on developments and collaboration and learn from experiences and results so far. All relevant stakeholders for each individual project will be invited to the audits, including the vulnerable target group when possible. The discussions will be semistructured, including topics on participation, outcomes, mechanisms, collaboration, and sustainability, but leaving room for discussions tailored to project-specific issues and developments. A timeline exercise [34-36] will be used to involve all participants in the discussion. A guideline for a semistructured group interview will be developed covering the aforementioned topics. Audits will be facilitated by a researcher, preferably the researcher who has led the *Effectenarena* session for this specific project. A second researcher or research assistant, present during the sessions, will draw up a short report, which will in turn be presented to the project leader. This person will be asked to judge how accurately the report reflects the group sessions.
2. Information collected through yearly rounds of interviews with all 46 projects leaders. Telephone interviews will be scheduled yearly with all project leaders. The interviews will be conducted by a member of the overall research team. Each interview will follow a predefined semistructured format, thereby ensuring that data are collected on results, participation, mechanisms, and sustainability, but leaving room for project-specific tailoring. Furthermore, the structure and the content of the interviews are dependent on the timing: the first round will focus more on participation and collaboration, whereas later rounds will focus more on results, mechanisms, and sustainability. Project leaders will be notified beforehand about the aim of the interview, the main topics, and the (anonymous) way in which the information will be used. Before the interview, their permission will be requested to record the conversation. All recordings will be transcribed, and both audio files and transcriptions will be stored at a secure site.
3. Information collected through project reporting forms. Project leaders will be regularly asked (approximately twice every year) to fill out a reporting form on developments and results within their projects. To minimize the research and accounting and administrative burden for project leaders, these forms will be drafted in collaboration with Fonds NutsOhra (FNO). FNO requires project leaders to regularly fill out reports, so combining these will be efficient.

Qualitative comparative analysis (QCA) [37-39] will be used to analyze the qualitative data from group interviews, interviews with project leaders, and project reporting forms. QCA is an analytic approach and a set of research tools that combines formalized cross-case comparisons with detailed within-case analysis [39]. QCA will be carried out using R QCA software [33].

### Step D: In-Depth, Realist Informed Case Studies

In our 46 projects, altogether, and for a substantial number of specific projects, outcomes and results are uncertain as well as emergent. That is why, following Glouberman and Zimmerman, we regard them as complex rather than simple or complicated situations [40], although this does not mean that the projects do not have simple or complicated components in them as well [31]. The complex nature of the projects requires an appropriate evaluation design. To deal with this complexity and the related importance of context [41], realist informed case studies will be executed in a fourth step (D). Unraveling mechanisms for health-related behavior change and improved perceived health is the main aim of these in-depth studies. The case studies will be designed to look at specific *approaches* or situations instead of studying specific projects or interventions. An approach or situation exists within projects; a project is often more than just this situation, for instance, building a relationship between a (care) professional and a family member or organizing a participatory session for a specific group of vulnerable families. In other words, the level of evaluation within the case studies is that of specific relevant situations rather than that of the complete intervention. This will ensure that the evaluation results are relevant to all stakeholders instead of just a few projects. Also, choosing some *projects* as a main subject of study might be discouraging for others, whereas choosing *approaches* may spark interest and learning for everyone and encourage far more projects. The case studies include 4 steps:

(1) Selection of 2 approaches and, per approach, 3 projects working with these approaches (a total of 6 projects). The selection of approaches is based on possibilities to study the approaches within the 46 projects and theoretical and societal relevance. Possible approaches are community participation, the role of health professionals in promoting healthy lifestyles, improving local networks, and so on. Project selection will be finalized in consultation with project leaders and program management. (2) Identification of possible causal pathways for each selected approach using a realist perspective. These causal pathways are often called C (Context), M (Mechanism), and O (Outcome) configurations [41,42]. Mechanisms are determinants of behavior that work to generate an intended or unintended outcome. In so doing, mechanisms depend strongly on context. Jagosh et al [43] refer to context as the *backdrop* of programs and research and can thus include cultural norms and history of a community, geographic location, the nature of existing social networks, and neighborhood infrastructure. Outcomes are the result of an interaction between mechanisms and context. Methodologies for the case studies include literature review, interviews, document analysis, and focus groups. (3) Identified causal pathways are translated into more abstract-level theories. Further field study, using focus groups, interviews, and participant observation, will test identified and alternative causal pathways. (4) Translation of findings into a more abstract level and possible refinement of the abstract-level theory. These realist case studies will provide information to answer the main research question on mechanisms (research question 2).

### Data Triangulation and Analysis

Steps A to D as described above ensure the collection of information on the overall impact of the program and on mechanisms of health-related behavior change at work in the projects. Using source triangulation (combining views from different stakeholders and perspectives) and method triangulation (combining qualitative and qualitative sources) can support better understanding. Data triangulation will combine the available information toward answering both research questions.

1. Results from the quantitative analysis will be compared with results from qualitative methods to provide answers on *impact*. The QCA as described above provides information that supports or contradicts the patterns derived from quantitative data analysis. Qualitative analysis will also include thematic coding and content analysis. The qualitative data will be used to complement, but also to question and test, the insights from quantitative analysis. In turn, the quantitative information will be used to inform further qualitative analysis.
2. Information from qualitative sources combined with realist informed case studies will provide insights into how the approaches within projects may bring about change: the *mechanisms* at play. A realist informed analysis, exploring and testing context mechanism outcome configurations such as those described above, is the basis. Mechanisms at work within approaches (eg, how does involving local professionals work in promoting physical activity) will be identified and tested. Further qualitative data, collected in addition to the case studies, may be used to further understand and explain these identified and tested mechanisms.

## Results

The overall evaluation project was funded in 2016. This study protocol included the design of at least 4 different studies. The results will hence provide information on (1) building and defining theories of change in health promotion practice, (2) mechanisms at work in promotion of healthy behavior among vulnerable families, (3) what works and what does not in professionals’ practices in health promotion among those vulnerable groups, and (4) what works and what does not in health promotion projects with a participatory approach. In addition, data will be collected on the overall effectiveness of the 46 initiatives. This will yield insights into possibilities for comparisons using diverse, quantitative, and qualitative data. The first data collection—the gathering and defining theories of change for each separate project—started in 2016, and data collection is currently ongoing. According to Dutch law, this study did not require formal ethics committee approval. However, special attention is paid in all activities to inform respondents and protect their privacy. All participants are provided with information about the purpose and contents of the research. Participation is voluntarily, and participants are able to withdraw from the study at any time for any reason. The collected data are treated confidentially and anonymously. Data analysis is currently under way, and the first results are expected to be submitted for publication in 2019.

## Discussion

### Opportunities

The 46 small-scale projects—which can be described as very diverse but with common principles—provide a unique opportunity for research on mechanisms in health promotion. They offer an extended range of relevant cases, instead of just one or two. To our knowledge, not many program evaluations have the same potential to provide such rich material on multiple cases in varying contexts. The availability of project-specific evaluation data provides the possibility to study the *impact* of different approaches with regard to changes in health-related behavior and perceived health. Similarities in strategies for health promotion as well as differences between projects enable such analysis. However, the diversity in the projects allows for a study protocol that also answers in greater depth questions of how specific health promotion approaches work, what we have called unraveling *mechanisms*. The multitude of contexts under study combined with various projects implementing similar approaches and activities potentially provides the opportunity to compare impact and mechanisms in interaction with contextual factors. Last but not least, the timing and the participatory approach applied in the overall evaluation enables all stakeholders to maximize learning throughout the 4 years of funding. Using a theory-based, complexity-sensitive approach that is predominantly realist informed, this study will also provide an opportunity to see whether combining assumptions from different approaches yields relevant information. This proposed combination of approaches in one evaluation design could theoretically open up black boxes while also elucidating more traditional measures of effectiveness.

### Challenges

In addition to the great opportunity provided, we acknowledge that the overall evaluation includes some challenges. The 3 important remarks are as follows: (1) the evaluation is shaped by the information available in the HFN program, (2) there is a difference between the program’s distal aim and the projects’ proximal focus, and (3) the possible weaknesses in the evaluation designs of the individual projects may lead to low-quality data on the overall level. We have briefly explained these remarks below.

First, FNO has laid out multiple guidelines as well as suggestions for project leaders to use in the design of their projects. Guidelines have been issued about the focus of the projects—health-related behavior or perceived health—and about target groups—socially vulnerable families. On the one hand, it seems that project leaders have been following these guidelines; all say they will focus on smoking cessation, the reduction of alcohol consumption, promoting healthy weight through feeding practices or exercise, or improving perceived health. On the other hand, regarding target groups, projects often seem to have been less compliant. Target groups are all classified as vulnerable families but range from single mothers with a low income or education to multiproblem households in specific urban areas. Also, the focus on health-related behavior may cause projects to ignore outcomes at the intermediate level. The diversity in target groups may complicate combined analyses of project data at the overall level. This means that, however rich the information offered by the program is, it may at times prove either too diverse or too focused for the researches to be able to analyze its effectiveness and processes at the higher program level. In this study, we addressed this issue by not only looking at effectiveness but also broadening the scope of the research to in-depth mechanisms of health promotion. In addition, we explored alternative ways of analysis to address effectiveness.

A related second limitation lies in the fact that, even though the programs aim to reduce health inequalities, a specific focus on health-related behavior among socially vulnerable families has been prescribed for the projects. Graham [17] has distinguished 3 approaches to reducing health inequalities. Targeted programs may improve the health of those in the worst socioeconomic position without making any effort to improve the health of those in higher socioeconomic positions. Other programs may target the health gap between low and high socioeconomic groups by improving the health of the poorest groups fastest. Another last approach Graham mentions is to explicitly address *gradients* in health inequalities [17,44]. Most HFN projects are designed to target specifically, and in several cases only, those in the lowest socioeconomic position. By not addressing the gradient, the projects may thus fail to improve the health of intermediate groups [44]. However, the information that this study may produce on mechanisms among the most vulnerable groups could be an important contribution to shaping future health promotion initiatives. As mechanisms operate in specific contexts—that is, for specific groups—results may even prove more valuable when restricted to a group, place, or time.

The gap in levels between the program’s aim and the projects’ focus may be seen as the difference between proximal and distal factors [28]: factors contributing to health that are on a level closer to the individual, such as behavior, and factors or differences that emerge at a level further away from the individual, such as societal inequalities. The program appears to build on the notion that positive outcomes on causes at the proximal level, behavioral changes, may indicate successful outcomes at the distal level: reducing health inequalities. Although the usage of the terminology of proximal and distal in an evaluation framework has certain advantages [45], especially in clarifying theoretical models, it also complicates matters [46]. One complication relevant to the program under study is that embracing the notions of proximal and distal may lead to considering 1 factor (in this case, the proximal: behavior change) as more important than others at the distal level in explaining and reducing health inequalities. Previous research indicates that, although behavior change is certainly related to changes in health inequalities, it is not considered the one most important explanation [20,47]. In-depth information on for whom, when, and where certain behavior change interventions work or not can, however, still contain valuable tools for the design of future health promotion interventions.

Finally, the proposed study design is not tailored to measure changes in inequalities per se in a traditional, experimental way. There are few possibilities to include control groups in the evaluation design. The projects’ geographical and target group boundaries are often vague and dynamic. Therefore, expected outcomes may appear at different levels and in a variety of sizes. In many projects, the project-specific evaluation design has been tailored to such dynamics and complexity, using quasi-experimental, nonexperimental, participatory, process-focused or mostly qualitative designs. The consequent limited possibilities for conducting a randomized experiment at the project level will complicate the aggregation of quantitative data at the higher level. We cannot change the fact that we have to work with a diverse range of data. Optimizing communication with project leaders and project researchers and starting off with an exploration of the possibilities for data aggregation, we still hope to make as much use as possible of the information available.

It is very valuable that the information from multiple relevant cases is combined and that all projects address the same proximal indicators for health. This evaluation enables us to study effectiveness in addition to mechanisms. Its timing, parallel to the implementation of the projects, allows for continuous learning by all stakeholders involved. The diversity in contexts and approaches additionally holds promise for the transferability of successful mechanisms, thereby informing future programs.

## Appendix

Multimedia Appendix 1 Peer-reviewer report from the Wageningen School of Social Sciences.

## Abbreviations

FNO: Fonds NutsOhra
HFN: Healthy Futures Nearby
QCA: Qualitative Comparative Analysis
WHO: World Health Organization
