# Supplementary material for: Evaluating the Healthy Futures Nearby Program: Protocol for Unraveling Mechanisms in Health-Related Behavior Change and Improving Perceived Health Among Socially Vulnerable Families in the Netherlands
Source: JMIR Res Protoc. 2019 Apr 2;8(4):e11305. doi: 10.2196/11305 (PMC6465974; doi:10.2196/11305)
Supplement: Multimedia Appendix 1 [file resprot_v8i4e11305_app1.pdf]

Review of PhD project "Reducing health inequalities through small scale health promotion projects among socially vulnerable families in the Netherlands; an overall, realist evaluation of the FNO programme 'Healthy Futures Nearby'" from the Health and Society Group of Wageningen University [LH-MK2]

Please, rate each aspect on a four-point scale between outstanding and poor by ticking the appropriate box. Please also explain your evaluation and give comments and suggestions how to improve the project.

**Criteria: quality and relevance**	**Outstanding**	**Good**	**Fair/some weaknesses**	**Poor**
Clarity of the proposal	☐	☒	☐	☐
Originality of the proposal	☒	☐	☐	☐
Use of existing knowledge	☐	☒	☐	☐
Methodological approach	☐	☐	☒	☐
Scientific relevance	☐	☒	☐	☐
Societal relevance	☒	☐	☐	☐

Please, provide detailed comments in support of the rating you have given above; please, also add comments that may help the applicant to improve the proposal if necessary

Comments: Because the study questions are very ambitious, the methodology is very ambitious. One wonders not about the quality of the proposed methods as much as the feasibility of completing the project in the allotted timeframe. Either research question or research question 2 would probably be a well-sized PhD project; tackling both will be very arduous. More on this in the section below.

**Criteria: feasibility**	**Outstanding**	**Good**	**Fair/some weaknesses**	**Poor**
Feasibility of the work plan	☐	☐	☒	☐
Appropriateness of the scope of the research	☐	☐	☒	☐
Expertise in the supervision team	☒	☐	☐	☐
Cooperation with others	☒	☐	☐	☐

Please, provide detailed comments in support of the rating you have given above; please, also add comments that may help the applicant to improve the proposal if necessary

Comments: It is comforting that the supervisors are very experienced. It is expected that they will guide the candidate through the thorny issues inherent in the study design. The overall feasibility is addressed above, In addition, the project depends on a lot of yet-to-be-collected data, the nature and quality of which is unknown. As much data will be gather by researchers other than the candidate herself, this is a somewhat risky situation. With so many actors involved directly and indirectly in this project, its complexity is of a rare nature for a PhD project. A lot could go wrong, and one misses a kind of SWOT analysis of the project plan. What could go wrong, and how will the project be adapted in response?<br><br>Parts of the project plan depend absolutely on the multi-level analysis of quantitative data. Yet the candidate writes that she will 'possibly' use MLWIN for multi-level modelling. This is disquietingly vague.<br><br>The actual methods of the qualitative analysis are also very vaguely presented. Will the analysis be mainly deductive, inductive, or a combination? The entire nature of the project hangs on such methodological considerations.<br><br>Having expressed these concerns, this review feels that the opportunity to comment on the project plan should have come well before the project was actually started. As the project is already well under way as of this writing, the possibility to help polish the plan has past by…

**Criteria: ethical issues**	**Yes**	**No**
Do you find ethical issues present in this proposal?	☒	☐
If yes, are the ethical issues handled satisfactorily?	☐	☒

Please, provide detailed comments that may help the applicant to improve the proposal if necessary

Comments: The requirement is that the issue of possible ethical <u>dilemmas</u> arising from this PhD research plan shall be addressed. This is not done to any extent.

**Overall assessment**		
Do you recommend revising the proposal?	☐ **Yes**	☒ **No**
If yes, which kind of revision do you recommend?	☐ **Major**	☒ **Minor**

Thank you very much for you co-operation and please return within two weeks to Wageningen School of Social Sciences: e-mail to: wass@wur.nl

Review of PhD project "Reducing health inequalities through small scale health promotion projects among socially vulnerable families in the Netherlands; an overall, realist evaluation of the FNO programme 'Healthy Futures Nearby'" from the Health and Society Group of Wageningen University [LH-MK3]

Please, rate each aspect on a four-point scale between outstanding and poor by ticking the appropriate box. Please also explain your evaluation and give comments and suggestions how to improve the project.

**Criteria: quality and relevance**	**Outstanding**	**Good**	**Fair/some weaknesses**	**Poor**
Clarity of the proposal	☒	☒	☐	☐
Originality of the proposal	☐	☒	☐	☐
Use of existing knowledge	☐	☒	☐	☐
Methodological approach	☐	☒	☒	☐
Scientific relevance	☐	☐	☒	☐
Societal relevance	☐	☒	☐	☐

Please, provide detailed comments in support of the rating you have given above; please, also add comments that may help the applicant to improve the proposal if necessary

Comments:the proposal is very clear and for sure societally relevant./ i am also quite convinced of its scientific relevance – this has, however, not been discussed. More could have been said about it explicitly – as the question as such has not been raised in the proposal at all. I also think more could and should have been said about realist theory – what it entails and also why it has been chosen above other approaches. I also would have liked to know more about what happens after the testing of various projects theories of changes and CMO configurations. How are the different theories then used to build a theory at higher level of scale? I also wonder how the effect of participation/empowerment and professional practices can be isolated and disentangled in terms of effect from the complexity of the projects, their measures and their mechanisms. I don't think the proposal needs to be revised to respond to this question but it may be useful to keep them in mind while further developing the project.

**Criteria: feasibility**	**Outstanding**	**Good**	**Fair/some weaknesses**	**Poor**
Feasibility of the work plan	☐	☒	☐	☐
Appropriateness of the scope of the research	☐	☒	☐	☐
Expertise in the supervision team	☐	☒	☐	☐
Cooperation with others	☐	☒	☐	☐

Please, provide detailed comments in support of the rating you have given above; please, also add comments that may help the applicant to improve the proposal if necessary

Comments: the project seems feasible

**Criteria: ethical issues**	**Yes**	**No**
Do you find ethical issues present in this proposal?	☒	☐
If yes, are the ethical issues handled satisfactorily?	☒	☐

Please, provide detailed comments that may help the applicant to improve the proposal if necessary

Comments:

**Overall assessment**		
Do you recommend revising the proposal?	☐ **Yes**	☒ **No**
If yes, which kind of revision do you recommend?	☐ **Major**	☐ **Minor**

Thank you very much for you co-operation and please return within two weeks to Wageningen School of Social Sciences: e-mail to: wass@wur.nl
